# Diversity of Bats in Contrasting Habitats of Hulu Terengganu Dipterocarp Forest and Setiu Wetland BRIS Forest with a Note on Preliminary Study of Vertical Stratification of Pteropodid Bats

**DOI:** 10.21315/tlsr2018.29.1.4

**Published:** 2018-03-02

**Authors:** Grace Pounsin, Nur Syahirah Wahab, Azuan Roslan, Muhamad Aidil Zahidin, Elizabeth Pesiu, Nur Aida Md Tamrin, M T Abdullah

**Affiliations:** 1 School of Marine and Environmental Sciences, Universiti Malaysia Terengganu, 21030, Kuala Terengganu, Terengganu, Malaysia; 2 Institute of Tropical Biodiversity and Sustainable Development, Universiti Malaysia Terengganu, 21030, Kuala Terengganu, Terengganu, Malaysia; 3 Department of Zoology, Faculty of Resource Science and Technology, Universiti Malaysia Sarawak, 94300, Kota Samarahan, Sarawak, Malaysia

**Keywords:** Bat, Diversity, Stratification, Dipterocarp Forest, BRIS Forest

## Abstract

A study of the bat diversity was conducted in Hulu Terengganu dipterocarp forest and Setiu Wetland Beach Ridges Interspersed with Swales (BRIS) forest in Terengganu, to study the species diversity, composition and stratification of fruit bats from the understorey to the forest canopy. Mist nets were set up at the understorey, sub-canopy and canopy layer while harp traps were set up at the understorey layer. We recorded 170 individuals from six families’ compromised 21 species from Hulu Terengganu dipterocarp forests and four species from Setiu Wetland BRIS forests throughout the sampling period. *Megaerops ecaudatus* and *Cynopterus brachyotis* were the most dominant species in Hulu Terengganu dipterocarp forest and Setiu Wetland BRIS forests. Our study also recorded two species with new distributional records for the east coast of Peninsular Malaysia, namely, *Rhinolophus chiewkweeae* and *Chaerephon johorensis* in Hulu Terengganu dipterocarp forests. Potential factors that might influence the results were in terms of the canopy covers, the structural complexity of canopy, food availability and spatial characteristics. This study was able to increase the knowledge on the species diversity and composition of bats in Hulu Terengganu dipterocarp forest and Setiu Wetland BRIS forest, thus, further aid in the effort of bat conservation in both areas.

## Abstract (ru)

Satu kajian kepelbagaian kelawar telah dijalankan di hutan dipterokarp Hulu Terengganu dan hutan *Beach Ridges Interspersed with Swales* (BRIS) tanah lembap Setiu di Terengganu untuk mengkaji kepelbagaian spesies, komposisi dan stratifikasi kelawar dari bawah hingga ke kanopi hutan. Jaring kelawar telah di pasang di bawah kanopi, sub-kanopi dan kanopi, manakala perangkap *harp* telah diletak pada lapisan bawah kanopi. Kami merekodkan 170 individu daripada enam keluarga dan 21 spesies dari hutan dipterokarp Hulu Terengganu dan lima spesies dari hutan BRIS tanah lembap Setiu sepanjang tempoh pensampelan. *Megaerops ecaudatus* dan *Cynopterus brachyotis* adalah spesies yang paling dominan dalam hutan dipterocarp Hulu Terengganu dan hutan BRIS tanah lembap Setiu. Kajian kami juga telah mencatatkan dua spesies yang mempunyai rekod penyebaran baru di pantai timur Malaysia, iaitu *Rhinolophus chiewkweeae* dan *Chaerephon johorensis* daripada hutan dipterokarp Hulu Terengganu. Faktor-faktor yang berpotensi mempengaruhi keputusan adalah dari segi litupan kanopi, kepelbagaian struktur kanopi, ketersediaan makanan dan ciri-ciri ruang. Kajian ini dapat meningkatkan pengetahuan mengenai kepelbagaian spesies dan komposisi kelawar di hutan dipterokarp Hulu Terengganu dan hutan BRIS tanah lembap Setiu. Dengan itu, kajian lanjut diperlukan dalam usaha pemuliharaan kelawar di kedua-dua kawasan tersebut.

## INTRODUCTION

Bats performed many significant ecological services and often regarded as a keystone species. In agricultural and natural ecosystems, they deliver some ecological regulating services such as pest suppression, seed dispersal and pollination that benefits human economically (Kunz *et al*. 2011). Globally, bats are the second most diverse mammals on earth after with 1,116 species currently, have been described (Kunz & Parsons 2009). Currently, in Malaysia, there are 133 species of bats in Malaysia (Kingston *et al.* 2012). In Peninsular Malaysia, the study on bats has been studied very well in Krau Wildlife Reserve, Peninsular Malaysian east-coast state of Pahang and also in Peninsular Malaysian west-coast states. However, the study of bats in Peninsular Malaysian east-coast despite being studied very well in Pahang, in other states namely Kelantan and Terengganu is still understudied. We also studied the vertical stratification of Pteropodid bats to increase the bat’s species checklist that forages in a higher level. The vertical stratification study of bats in Malaysia has been conducted by Francis (1994), Zubaid (1994), Abdullah and Hall (1997), and Hodgkison *et al.* (2004). The study on bats ecology in Malaysia has been concentrated at ground level due to easy deployments of mist nets and harp traps. The study on vertical stratification on bats is still understudied in Malaysia with only five known published information.

This study has been conducted in Hulu Terengganu district which is part of Central Forest Spine (CFS) Project and Setiu Wetland in Setiu district. The habitat and forest types in these two study sites are different and contrasting where Hulu Terengganu is a dipterocarp forest and Setiu Wetland consisting of various forest types. Currently, there are some established forest reserves (FR) in Terengganu, namely, Lata Belatan FR, Lata Tembakah FR, Lata Paying FR, Bukit Kesing FR, Sekayu FR, Chemerong FR, Jambu Bongkok FR, Rimba Bandar Bukit Bauk FR, Air Menderu FR, Rasau Kertih FR, and Besul FR. Other than that, dipterocarp forests in Hulu Terengganu also serve as a part of the CFS, which is the seventh, out of 17 linkages in Peninsular Malaysia. The CFS is one of the government efforts to create a broad adjoining forest corridor through the combination of interconnecting forests in 17 habitat linkages (Hedges *et al*. 2013). Due to the discovery of unique floral and faunal composition in Setiu Wetland (Tamblyn *et al.* 2006; Pesiu *et al.* 2015), this area is proposed to be the first state park of Terengganu along with the motion of establishing Tasik Kenyir in Hulu Terengganu and its surrounding forests as a Geopark (Che Aziz *et al.* 2015). Perhaps the information from this study will assist the government and non-governmental agencies in conservation planning and decision making.

## MATERIALS AND METHODS

### Description of the Study Area and Sampling Site

**Figure 1:**
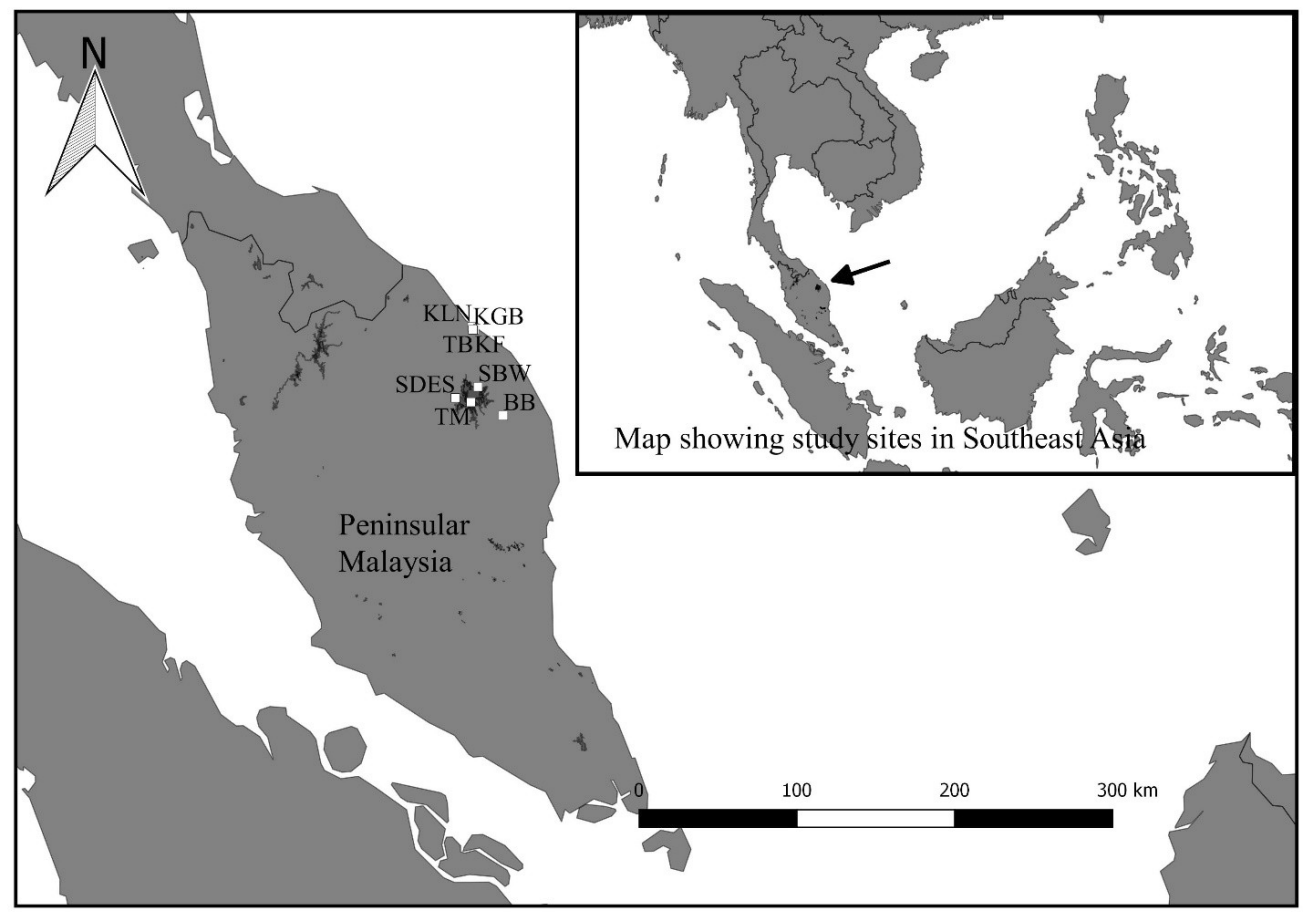
Study sites of bats in Terengganu. Hulu Terengganu dipterocarp forest: SBW-Sungai Buweh Waterfall, SDES- Sungai Deka Elephant Sanctuary, TM- Tanjung Mentong and BB- Belukar Bukit. Setiu Wetland BRIS forest: KLN- Kampung Limau Nipis, KF-Kampung Fikri, KGB- Kampung Gong Batu and TB- Tasik Berombak.

The sampling period was done from January to March 2015. The sampling sites and sampling duration within those study areas are as shown in Figure 1 and described in Table 1.

A vegetation survey was done following Soepadmo (1971) on the plant diversity in the study areas. The vegetation surveys were done in Hulu Terengganu dipterocarp forests (Appendix A) and Setiu Wetland BRIS forests (Figure 1; Appendix B). The most of the common tree species observed were recorded. Descriptions of the habitat types and their coordinates are listed as in Table 1.

### Field Methods

Due to the limited resources and difficulty of canopy access and deployment, only eight mist nets and three four-banks harp traps were deployed in the understorey level whereby two to five mist nets were set up at the canopy layer for six consecutive nights. Each mist nets and harp traps below the canopy layer were set up at various locations, such as, across trails and water streams which were believed to be the flyways for bats (Kunz & Kurta 1988; Khan *et al*. 2007). The understorey mist nets were relocated after two days to cover more area and increase the chances of catching more individuals and species (Mohd-Hanif *et al*. 2015) and to prevent the “net shyness” (Kunz & Kurta 1988). The canopy mist nets were deployed by using two methods; sets of scaffolding towers with an approximate of 12 m height from the ground floor level and another using rope and pulley systems. All of the mist nets and harp traps were checked regularly with an interval of 15–30 minutes after dusk until 9.00 pm and were continued in the following mornings from 5.00 am to 6.30 am.

**Figure 2:**
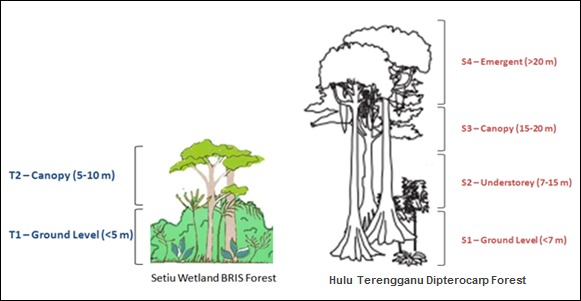
The canopy stratification level in Hulu Terengganu dipterocarp forest and Setiu Wetland BRIS forest.

The stratification of Hulu Terengganu dipterocarp forest followed the method by Abdullah and Hall (1997) as; forest floor (S1: <7 m), understorey level (S2: 7 to 15 m), canopy level (S3: 15 to 20 m) and emergent (S4: >20 m; Figure 2). Meanwhile, the stratification of Setiu Wetland BRIS forest can be generalised into ground level (T1: <5 m) and canopy level (T2: 5 to 10 m; Figure 2) due to the relatively lower height as compared to Hulu Terengganu dipterocarp forest.

The species identification followed the keys by Francis (2008) and Kingston *et al.* (2009). The morphological measurements were taken using digital vernier calliper. The weight for each individual was weighted using a spring balance and selected species were photographed for future references. Representative species (maximum of five individuals for each species) were preserved in 70% ethanol solution and deposited in the Museum of Zoology Kenyir (MZK), Universiti Malaysia Terengganu (UMT) for future references.

Paleontological Statistic (PAST) v2.17 software (Hammer *et al*. 2012) was used to calculate Shannon’s diversity index. To perform diversity comparison with unequal sampling effort among sampling sites, we used rarefaction curve constructed by using EcoSim v1.2d software (Entsminger 2014) and Microsoft Excel. We also constructed species accumulation curve using EcoSim software. In addition, a taxonomic list of bats was compared with previous studies done within Hulu Terengganu dipterocarp forest and Setiu Wetland BRIS forest.

## RESULTS

### Vegetation Survey

Following the classification by Saw (2010), several characteristics of the forest formations and vegetation were gathered in order to describe the forests of Hulu Terengganu. Generally, Hulu Terengganu is occupied by lowland evergreen rain forest with per-humid (a seasonal) climate having elevations ranging from sea level to 1,200 m above sea level. The lowland evergreen rainforest has three tree layer characteristics, mainly upper layer with emergent trees as tall as or greater than 70 m, the main stratum at about 24 m until 36 m and a lower layer with smaller, shade-tolerant trees and immature trees. Meanwhile, the forest floor or ground vegetation often sparsely occupied by small trees, shrubs, herbs and understorey palms (Saw 2010).

In Peninsular Malaysia, the lowland evergreen rainforests can be described further into three sub-forest types based on the changes in species composition that occur with altitude (Symington 1943; Wyatt-Smith 1963; Saw 2010). The three sub-forest types, namely lowland dipterocarp forest (<300 m), hill dipterocarp forests (300–750 m) and upper hill dipterocarp forests (750–1200 m) and these can be found in Hulu Terengganu. However, in this research, more evidence was found on the lowland dipterocarp forest giving an indication that the study site is located within the extensive stands of lowland dipterocarp forests. Moving into the species compositions, the common tree species in lowland dipterocarp forests of Hulu Terengganu include *Hopea dyeri*, *Shorea leprosula*, and *Dipterocarpus grandiflorus* from the major timber family, Dipterocarpaceae. The other tree species include *Baccaurea parviflora* and *Croton laevifolius* (Euphorbiaceae); *Pometia pinnata* (Sapindaceae), *Monocarpia marginalis* (Annonaceae), and *Saraca thaipingensis* (Caesalpiniaceae) (Pesiu *et al*. 2015). This description is also consistent to the distributions of the tree species by Turner (1995).

Setiu Wetland is well-known for its unique soil formation called Beach Ridges Intersped with Swales (BRIS). Recent studies by Jamilah *et al*. (2013) on the natural vegetation of BRIS soil ecosystem on the coastal dune of Terengganu has described three distinct natural vegetation formations on the BRIS soil ecosystem as lowland mixed dipterocarp forest, melaleuca swamp and heath vegetation. Our extensive study has been done in some parts of Setiu Wetland revealing similar vegetation types that made up the land. Small patches of peat swamp forest, mangrove forests, and beach vegetation occupied the land of Setiu. The mangrove forests and beach vegetation generally made up the coastal framework of Setiu Wetland with small patches of beach vegetation interval with the mangrove forests decorating the continuous coastal region along Setiu Wetland. The mangrove forest generally dominated by species from family Rhizophoraceae. Common species that can be found in mangrove forest are *Rhizophora apiculata* (Rhizophoraceae), *Heritiera littoralis* (Malvaceae), *Pouteria obovata* (Sapotaceae), *Excoecaria agallocha* (Euphorbiaceae), and *Hibiscus tiliaceus* (Malvaceae) (Pesiu *et al*. 2015).

Meanwhile, predominant species found with beach vegetation was *Casuarina equisetifolia* (Casuarinaceae). Other tree species, including *Terminalia catappa* (Combretaceae), *Pandanus odoratissimus* (Pandanaceae) and *Cycas litoralis* were commonly found. Other vegetation types that can be found in Setiu is the peat swamp forest. This type of forest is not too notable due to its adaptations on permanently waterlogged site; often hidden by the large scale of heath vegetation and *Melaleuca* swamp forests. However, the tree species within this forests are distinctive and easily identified. Some of the frequently encountered species were *Alstonia spatula* (Apocyaceae), *Campnosperma* sp. (Anacardiaceae), *Macaranga hypoleuca* (Euphorbiaceae), *Cratoxylum arborescens* (Hypericaceae) and *Ilex cymosa* (Aquifoliaceae) found to be scattered throughout the forests (Pesiu *et al*. 2015).

### Chiropteran Survey

A total of 170 individuals of chiropterans representing 21 species from six families were recorded in Hulu Terengganu dipterocarp forests and Setiu Wetland BRIS forests (Appendix C). From the total of species captured, eight species were frugivorous and the rest were insectivorous. We identified Sungai Buweh Waterfall as sampling site with the highest recorded species (14 species), whereas Kampung Limau Nipis site with the least recorded species (one species). The highest number of individuals recorded was at Sungai Buweh Waterfall (45 individuals) and the least, was at Kampung Fikri (three individuals). *Cynopterus brachyotis* was encountered in all study sites and were the most abundant in Setiu Wetland BRIS forest. Moreover, *Megaerops ecaudatus* was the dominant species in Hulu Terengganu dipterocarp forest. Our study also highlighted the two new distributional record species in Sungai Buweh Waterfall and Belukar Bukit, which were *Rhinolophus chiewkweeae* (Morni *et al.* 2016) and *Chaerephon johorensis* (Roslan *et al.* 2016).

The taxonomic diversity of chiropterans based on stratification level shows that pteropodid bats utilised each forest stratum in Hulu Terengganu dipterocarp forests and Setiu Wetland BRIS forests. In Hulu Terengganu dipterocarp forest, we netted five species in the canopy layer of Belukar Bukit forest, namely, *Penthetor lucasi* (20.34 m height) *M. ecaudatus*, *C. johorensis* and *Megaderma spasma* (20.65 m); and *Balionycteris maculata* (22.65 m). Only *C. brachyotis* was netted at canopy level (T2) in Setiu Wetland forests at 12 m from the ground floor in Tasik Berombak. The rest of the species dominantly occurred on a much lower elevation in both study areas.

**Figure 3:**
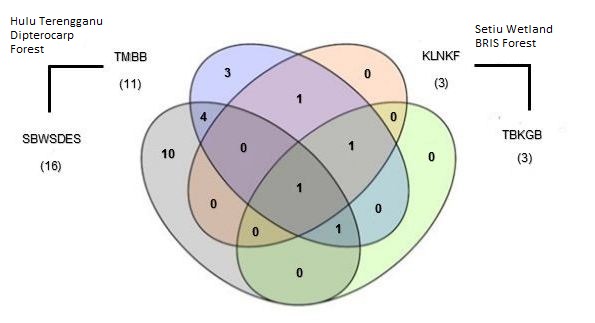
Venn diagram showing numbers of unique and shared species between Hulu Terengganu dipterocarp forest and Setiu Wetland BRIS forest. Sampling sites were pooled to represent each ecosystem of dipterocarp and BRIS forest. The number of species is shown in each of the subsets and the total number of species for each sampling sites are given in parentheses. SBWSDES is combination of Sungai Buweh Waterfall and Sungai Deka Elephant Sanctuary; TMBB- combination of Tanjong Mentong and Belukar Bukit; KLNKF- combination of Kampung Limau Nipis and Kampung Fikri; TBKGB- combination of Kampung Gong Batu and Tasik Berombak.

Figure 3 shows unique and shared species between two contrasting ecosystems; Hulu Terengganu dipterocarp forest and Setiu Wetland forest. The insectivorous bats were captured in ground level except for *C. johorensis*. Within the Family Rhinolophidae*, R. affinis, R. chiewkweeae,* and *R. luctus* are confined to the dipterocarp forest while *R. lepidus* is found in both ecosystems. Meanwhile, *C. brachyotis* was observed to be present in all sampling sites of two contrasting ecosystems in Terengganu.

**Figure 4:**
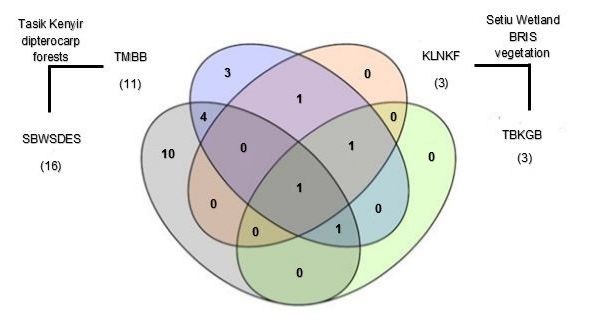
Venn diagram showing numbers of unique and shared species between forest strata; understorey, sub-canopy, canopy and emergent layer in Hulu Terengganu dipterocarp forest and Setiu Wetland BRIS forest. The number of species is shown in each of the subsets and the total number of species for each sampling sites are given in parentheses.

Figure 4 shows unique and shared species between four forest strata in both Hulu Terengganu dipterocarp forest and Setiu Wetland BRIS forest. The understorey layer recorded the highest number of species followed by the canopy layer and sub-canopy layer. Species that are shared between the understorey and canopy layer showed that those species, namely, *B. maculata*, *C. brachyotis*, *P. lucasi* and *M. spasma* can be found in both lower and upper elevations. Meanwhile, *C. brachyotis* was found in the understorey, sub-canopy and canopy layer.

**Figure 5:**
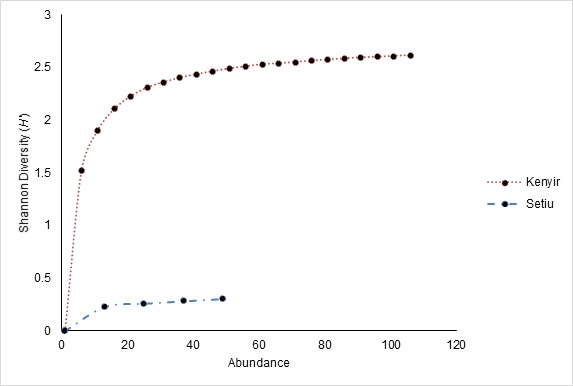
The individual rarefaction curve of bats in Hulu Terengganu dipterocarp forest and Setiu Wetland BRIS forest. The highest diversity of both sites after rarefaction curve analysis 2.46 in Hulu Terengganu dipterocarp forest.

Sungai Buweh Waterfall has the highest diversity compared to the other sites with the highest number of Shannon index is 2.123 (Table 2). Meanwhile, Kampung Limau Nipis has the lowest diversity (Shannon index = 0.000). Overall, Hulu Terengganu dipterocarp forest hold the highest diversity of bats (Shannon index = 2.619) compared to Setiu Wetland BRIS forest (Shannon index = 0.314; Table 3). The individual rarefaction curve of bats in Hulu Terengganu dipterocarp forest and Setiu Wetland BRIS forests is shown in Figure 5. The 95% confidence intervals showed that the species richness between the two areas is significantly different.

## DISCUSSION

From the result, the bat diversity of Hulu Terengganu dipterocarp forest is higher than Setiu Wetland BRIS forest. There were many factors that contributed to the higher number of bats in Hulu Terengganu dipterocarp forest. Dipterocarp forest holds up more niches for higher numbers of unique species compositions (Hill & Hill 2001; Bruhl *et al*. 2003; Okuda *et al*. 2003; Podong & Poolsiri 2013) due to a denser forest area with higher structural diversity, and higher food availability (Molles 2005). Meanwhile, Setiu Wetland BRIS forest recorded the lower diversity of bats due to the forest clearance by anthropogenic activities such as human settlements, agriculture, aquaculture, and land conversion into palm oil plantations that caused forest fragmentation (Tamblyn *et al*. 2006; Mohd Azmi 2014; Suratman *et al*. 2014). Fragmentation followed by deforestation had caused serious impact towards the loss of bats habitats in Southeast Asia (Lane *et al*. 2006; Sodhi & Brook 2006). Fukuda *et al*. (2009) reported that chiropterans were one of the most susceptible taxa affected by anthropogenic activities. However, the high abundance of *C. brachyotis* in Setiu wetland forest shows that this species is able to thrive in most areas including those in the disturbed vicinity. This species is a strong flyer and is cosmopolitan species (Abdullah, 2003). This species has cryptic forms, the small-sized and the large-sized where the small-sized *C. brachyotis* inhabit the forested area whereas the large-sized *C. brachyotis* preferring the open areas (Abdullah *et al*. 2010; Abdullah, 2003).

The results of this study were compared with previous studies conducted in various habitats in Hulu Terengganu dipterocarp forest and Setiu Wetland BRIS Forest and Jambu Bongkok FR BRIS Forest (Appendix D). The present study done in Hulu Terengganu recorded the higher number of bat species compared to Norfarhana *et al*. (2015);Shahrul Anuar & Mohd Hifni (2001), Nor Zalipah *et al.* (2010) and Nor Zalipah *et al.* (2015) due to the longer sampling periods, covering more areas, and the usage of canopy mist nets. The effective usage of canopy mist nets resulting more capture on species that commonly forage in a higher level such as *R. amplexicaudatus, E. spelaea,* and *M. ecaudatus.* Francis (1994) noted that the capture rates for the bats above the ground level are more than 100 times. This study also recorded the new distributional records namely *R. chiewkweeae* and *C. johorensis.* The *R. chiewkweeae* were captured in Sungai Buweh Waterfall forest marked the seventh records in Malaysia (Morni *et al.* 2016) after Lubuk Sembilang, Langkawi Island, and Ulu Muda FR in Kedah, Asahan FR in Melaka, Gunung Ledang and Labis FR in Johor, and Wang Kelian State Park in Perlis (Yoshiyuki & Lim 2005; Jayaraj *et al*. 2011). Meanwhile, *C. johorensis* was the first record in Terengganu in Belukar Bukit forest and forth record in Malaysia (Roslan *et al*. 2016) after Hutan Lipur Air Terjun Batu Hampar, Kedah (Jayaraj *et al*. 2013) and Krau Wildlife Reserve, Pahang, Malaysia (Francis 2008).

Meanwhile, for the vertical stratification of fruit bats, the abundance of fruit bats in sub canopy level and canopy level in Hulu Terengganu dipterocarp forest is comparable with the study done by Francis (1994); Zubaid (1994); Abdullah & Hall (1997) and Hodgkison *et al.* (2004). High distribution of species from family Pteropodidae at the canopy layer shows that higher food source is available and the tropical rain forests’ canopy layers provide more fruits to animals such as bats (Harrison 1962; Francis 1994; Abdullah & Hall 1997; Mohd-Azlan *et al*. 2000). Although we used mist nets to capture fruit bats, we also managed to capture insectivorous bats in canopy level namely *C. johorensis.* This species is known to forage at the canopy level (Francis 2008). In Setiu Wetland BRIS forest, there was only *C. brachyotis* captured at the height of more than 10 m, the other species were captured at the height range of less than 10 m. This is because the forest in Setiu Wetland BRIS forest has been logged therefore many species that were generally foraging in canopy such as *E. spelaea* can be found at ground level.

## CONCLUSION

The result of the bat diversity in contrasting ecosystems shows that the species diversity of bats in Terengganu is still understudied. There are still many areas need to be explored to obtain comprehensive bat data. More species recorded in Hulu Terengganu dipterocarp forest were due to the higher structural complexity of the vegetation that fulfills the needs of the bat’s niche compared to the less complex composition of Setiu Wetland BRIS forest. Particular species that were found and dominated certain forest strata implies that the species adaptations complemented the structure and features of the certain strata they inhabit. For improvement in doing further research, sampling period and the number of nets deployed particularly at canopy levels should be increased to get more recorded species and number of bats. Lunar phase needs to be avoided during the sampling period because the nocturnal animals are passive during the full moon phase. The bat detector and camera traps can be deployed at canopy level to detect more bat species.

## Figures and Tables

**Figure 6:**
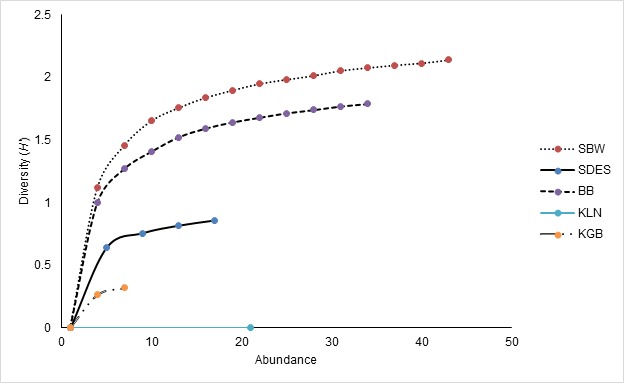
The individual rarefaction curve of each study sites at Hulu Terengganu dipterocarp forest and Setiu Wetland BRIS forests. Study sites at and Tanjung Mentong (Hulu Terengganu dipterocarp forest), and Kampung Fikri and Tasik Berombak (Setiu Wetland BRIS forest) are excluded due to the low number of species captured. The highest species diversity was observed in Sungai Buweh Waterfall. SBW- Sungai Buweh Waterfall, SDES- Sungai Deka Elephant Sanctuary, BB- Belukar Bukit, KLN- Kampung Limau Nipis, KGB- Kampung Gong Batu.

**Figure 7:**
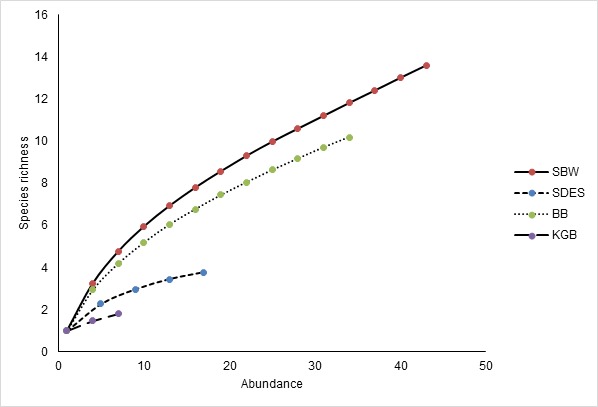
Species accumulation curve showing the species increasing trend. Study sites at Tanjung Mentong (Hulu Terengganu dipterocarp forest) and, Kampung Limau Nipis, Kampung Fikri and Tasik Berombak (Setiu Wetland BRIS forest) are excluded due to the low number of species captured. SBW- Sungai Buweh Waterfall, SDES- Sungai Deka Elephant Sanctuary, BB- Belukar Bukit and KGB- Kampung Gong Batu.

**Table 1:** List of study areas, forest types, date of studies and locations.

Study Areas	Forest Types	Date	Locations
**Hulu Terengganu dipterocarp forest**			
Sungai Buweh Waterfall	Lowland riparian forest	19 to 23 January 2015	N 05° 08′52″ E 102° 46′00″
Sungai Deka Elephant Sanctuary	Lowland and old secondary forest	9 to 12 February 2015	N 05° 02′58″ E 102° 33′57″
Tanjung Mentong	Hill dipterocarp forest	7 to 13 March 2015	N 05° 00′39″ E 102° 42′13″
Belukar Bukit	Old secondary forest	15 to 21 May 2015	N 04° 53′25″ E 102° 59′ 33″
**Setiu Wetland BRIS forest**			
Kampung Limau Nipis	BRIS forest	21 to 27 February 2015	N 05° 40′58″ E 102° 42′58″
Kampung Fikri	BRIS forest	23 to 29 March 2015	N 05° 39′19″ E 102° 44′08″
Kampung Gong Batu	BRIS forest	30 March 5 April 2015	N 05° 39′23″ E 102° 43′18″
Tasik Berombak	BRIS forest	7 to 13 March 2015	N 05° 39′19″ E 102° 43′37″

BRIS = Beach Ridges Interspersed with Swales

**Table 2:** Diversity and species richness of chiropteran in each study sites.

Station	Dipterocarp Forest				Setiu Wetland BRIS Forest			

	SBW	SDES	TM	BB	KLN	KF	KGB	TB
n	45	21	5	39	40	3	9	9
Shannon Index	2.123	0.887	0.500	1.817	0.000	0.637	0.349	0.562
Species Richness	3.415	0.985	0.621	2.730	0.000	0.910	0.455	0.481

SBW: Sungai Bewah Waterfall; SDES: Sungai Deka Elephant Sanctuary; TM: Tanjung Mentong; BB: Belukar Bukit; KLN: Kampung Limau Nipis; KF: Kampung Fikri; KGB: Kampung Gong Batu; TB: Tasik Berombak

**Table 3:** Diversity and species richness of bats in Hulu Terengganu dipterocarp forest and Setiu Wetland BRIS forest.

Station	Hulu Terengganu dipterocarp forest	Setiu Wetland BRIS forest
n	110	60
Shannon Index	2.619	0.314

n = number of individuals

## APPENDICES

### Appendix A

Photographs of the habitats in Tasik Kenyir. (a) Sungai Buweh Waterfall, (b) Belukar Bukit, (c) Sungai Deka Elephant Sanctuary, (d) Tanjung Mentong.

### Appendix B

Photographs of the habitats in Setiu Wetland. (e) Kampung Fikri, (f) Kampung Gong Batu, (g) Tasik Berombak, (h) Kampung Limau Nipis.

### Appendix C

Taxonomic compositions, stratification and relative abundance in parentheses of bats in Hulu Terengganu dipterocarp forest and Setiu Wetland BRIS forest in this study.

**Table:**

	Family	Hulu Terengganu dipterocarp forests								Setiu Wetland (BRIS Forest)							
	
	Species	SBW		SDES		TM		BB		KLN		KF		KGB		TB	
		n (RA)	SC	n (RA)	SC	n (RA)	sc	n (RA)	SC	n (RA)	SC	n (RA)	SC	n (RA)	SC	n (RA)	SC
	**Pteropodidae**																
1	*Balionycteris maculata*	0	-	0	-	0	-	4 (0.024)	S1	0	-	0	-	0	-	2 (0.012)	T1
2	*Cynopterus brachyotis*	1 (0.006)	S1	3 (0.018)	S1.S2	1 (0.006)	S2	7(0.041)	S1.S3	40 (0.235)	T1.T2	2(0.012)	T1	8 (0.047)	T1.T2	6 (0.035)	T1.T2
3	*Cynopterus horsfieldii*	0	-	0	-	0	-	1 (0.006)	S1	0	-	0	-	0	-	0	-
4	*Eonycteris spelaea*	0	-	0	-	0	-	1 (0.006)	S1	0	-	1 (0.006)	T1	0	-	0	-
5	*Macroglossus minimus*	1 (0.006)	S1	0	-	0	-	1 (0.006)	S1	0	-	0	-	0	-	0	-
6	*Macroglossus sobrinus*	0	-	0	-	0	-	0	S1	0	-	0	-	0	-	0	-
7	*Megaerops ecaudatus*	1 (0.006)	S3	0	-	0	-	2 (0.012)	S1	0	-	0	-	0	-	0	-
8	*Penthetor lucasi*	1 (0.006)	S1	0	-	0	-	17 (0.100)	S3	0	-	0	-	0	-	0	-
	**Megadermatidae**								S3								
9	*Megaderma spasma*	0	-	1 (0.006)	S1	4(0.024)	S1	1 (0.006)	S1	0	-	0	-	0	-	0	-
	**Rhinolophidae**																
10	*Rhinolophus affinis*	4 (0.024)	S1	0	-	0	-	3 (0.018)	S1	0	-	0	-	0	-	0	-
11	*Rhinolophus chiewkweeae*	4 (0.024)	S1	0	-	0	-	0	-	0	-	0	-	0	-	0	-
12	*Rhinolophus lepidus*	1 (0.006)	S1	0	-	0	-	1 (0.006)	S1	0	-	0	-	1 (0.006)	T1	0	-
13	*Rhinolophus luctus*	1 (0.006)	S1	0	-	0	-	0	-	0	-	0	-	0	-	0	-
	**Hipposideridae**																
14	*Hipposideros bicolor*	1 (0.006)	S1	0	-	0	-	0	-	0	-	0	-	0	-	0	-
15	*Hipposideros cervinus*	10 (0.059)	S1	0	-	0	-	0	-	0	-	0	-	0	-	0	-
16	*Hipposideros diadema*	1 (0.006)	S1	2 (0.012)	S1	0	-	0	-	0	-	0	-	0	-	0	-
17	*Hipposideros dyacorum*	6 (0.035)	S1	0	-	0	-	0	-	0	-	0	-	0	-	0	-
18	*Hipposideros larvatus*	12 (0.071)	S1	0	-	0	-	0	-	0	-	0	-	0	-	0	
	**Vespertillionidae**																
19	*Kerivoula pellucida*	1 (0.006)	S1	0	-	0	-	0	-	0	-	0	-	0	-	0	-
20	*Myotis ridleyi*	0	-	15 (0.088)	S1	0	-	0	-	0	-	0	-	0	-	0	-
	**Molossidae**																
21	*Chaerephon johorensis*	0		0	-	0	-	1 (0.006)	S3	0	-	0	-	0	-	0	-

	**Number of individuals**	45		21		5		39		40		3		9		8	
	**Number of species**	14		4		2		11		1		2		2		3	
	**Number of families**	4		4		2		4		1		1		2		2	
	**Sampling effort**	52		32		40		78		48		40		40		40	
	**Capture rate**	0.865		0.656		0.125		0.5		0.833		0.075		0.225		0.2	
	**Diversity index**	2.147		0.887		0.673		1.817		0		0.637		0.349		0.562	

n = number; RA = relative abundance; SBW = Sungai Buweh Waterfall, SDES = Sungai Deka Elephant Sanctuary, TM = Tanjong Mentong, BB = Belukar Bukit, KLN = Kampung Limau Nipis, KF = Kampung Fikri, KGB = Kampung Gong Batu,TB = Tasik Berombak; SC = Strata Class; S1: <7 m, S2: 7–15 m, S3:15–30 m; S4: >30 m (Abdullah and Hall, 1997); T1: <10 m and T2:10–16 m

### Appendix D

Comparison of taxonomic list of species recorded in this study with previous studies in Hulu Terengganu dipterocarp forest and Setiu Wetland BRIS forest.

**Table:**

No.	Family	Hulu Terengganu				Setiu Wetland			Jambu Bongkok FR	IUCN 2013
	
		Dipterocarp forest				BRIS forest				
	
	Species	This study	Norfarhana *et al*. (2015)	Shahrul Anuar & Mohd Hifi (2001)	Jayaraj *et al.* (2015)	This study	Tamblyn *et al.* (2006)	Nor-Zalipah *et al* (2015)	Nor-Zalipah *et al.* (2010)	
	**Pteropodidae**									
1	*Rousettus amplexicaudatus*	−	+	−	−	−	−	+	−	LC
2	*Cynopterus brachyotis*	+	+	+	+	+	+	+	−	LC
3	*Cynopterus horsfieldii*	+	−	+	−	−	−	+	−	LC
4	*Penthetor lucasi*	+	+	+	−	−	+	−	−	LC
5	*Megaerops ecaudatus*	+	+	−	−	−	−	−	−	LC
6	*Dyacopterus spadiceus*	−	−	−	−	−	+	−	−	NT
7	*Chironax melanocephalus*	−	−	−	−	−	+	−	−	LC
8	*Balionycteris maculata*	+	+	+	−	+	+	−	−	LC
9	*Eonycteris spelaea*	+	+	+	−	+	+	+	−	LC
10	*Maoroglossus minimus*	+	+	+	−	−	−	−	−	LC
11	*Macroglossus sobrinus*	+			−	−	+	−	−	LC
	**Nycteridae**				−					
12	*Nycteris tragata*			−	+					
	**Megadermatidae**				−					
13	*Megaderma lyra*	−	+	−	−	−	−	−	−	LC
14	*Megaderma spasma*	+	+	−	−	−	+	−	−	LC
	**Rhinolophidae**				−					
15	*Rhinolophus trifoliatus*	−	−	−	−	−	−	−	+	LC
16	*Rhinolophus sedulus*	−	+	−	−	−	−	−	−	NT
17	*Rhinolophus luctus*	+	−	−	−	−	−	−	−	LC
18	*Rhinolophus marshalli*	−	−	−	−	−	+	−	−	LC
19	*Rhinolophus acuminatus*	−	−	−	−	−	−	−	−	LC
20	*Rhinolophus lepidus*	+	−	−	−	+	−	−	+	LC
21	*Rhinolophus affinis*	+	−	−	+	−	−	−	−	LC
22	*Rhinolophus robinsoni*	−	−	−	+	−	−	−	−	NT
23	*Rhinolophus stheno*	−	−	−	+	−	−	−	−	LC
24	*Rhinolophus creaghii*	−	−	−	−	−	+	−	−	LC
25	*Rhinolophus chiewkweeae*	+	−	−	−	−	−	−	−	NE
	**Hipposideridae**									
26	*Hipposideros bicolor*	+	−	−	+	−	−	−	−	LC
27	*Hipposideros armiger*	−	−	−	−	−	−	−	−	LC
28	*Hipposideros cineraceus*	−	+	−	+	−	−	−	−	LC
29	*Hipposideros dyacorum*	+		−	+	−	−	−	−	LC
30	*Hipposideros cervinus*	+	−	−	+	−	+	−	−	LC
31	*Hipposideros galeritus*			−	+					LC
32	*Hipposideros larvatus*	+	+	−	+	−	−	−	−	LC
33	*Hipposideros diadema*	+	+	−	+	−	−	−	−	LC
34	*Hipposideros armiger*			−	+	−	−	−	−	LC
	**Vespertilionidae**									
35	*Myotis muricola*	−	−	−	−	−	−	−	+	LC
36	*Myotis ridleyi*	+	+	−	−	−	−	−	−	NT
37	*Myotis horsfieldii*	−	+	−	+	−	−	−	−	LC
38	*Myotis siligorensis*	−	+	−	−	−	−	−	−	LC
39	*Scotophilus kuhlii*	−	−	−	−	−	+	−	+	LC
40	*Tylonycteris pachypus*	−	+	−	−	−	−	−	−	LC
41	*Munna aenea*	−	−	−	−	−	−	−	+	VU
42	*Kerivoula minuta*			−	+					NT
43	*Kerivoula intermedia*	−	−	−	−	−	−	−	+	NT
44	*Kerivoula pellucida*	+	−	−	−	−	−	−	−	LC
45	*Miniopterus magnater*	−	−	−	−	−	+	−	−	LC
46	*Miniopterus medius*	−	−	−	−	−	−	−	+	LC
	**Mollosidae**				−					
47	*Chaerephon johorensis*	+	−	−	−	−	−	−	−	VU

	**Number of families**	6	5	1	5	2	6	1	2	
	**Number of species****	21	17	6	15	4	14	4	7	
	**Number of individuals****	110	56	n.a.	30	60	43	221	27	
	**Sampling effort/nights**	312	n.a.	n.a.	n.a.	312	n.a.	n.a.	33	
	**Capture rate**	0.35	n.a.	n.a.	n.a.	0.19	n.a.	n.a.	0.82	

** The Unidentified species were excuded from the taxonomic list; IUCN = International Union for Conservation of Nature; NE = Not Evaluated, LC = Least Concern, VU = Valnerable, NT = Near Thrated; n.a = data not available
